# A study on otoacoustic emissions and supression effects in patients with Vitiligo

**DOI:** 10.1016/S1808-8694(15)30841-7

**Published:** 2015-10-18

**Authors:** Rosanna Mariangela Giaffredo Angrisani, Marisa Frasson de Azevedo, Liliane Desgualdo Pereira, Celso Lopes, Michele Vargas Garcia

**Affiliations:** 1 Speech therapist.; 2 Doctorate, adjunct professor, Speech Therapy Department, Universidade Federal de Sao Paulo - Escola Paulista de Medicina.; 3 Doctorate, associate professor, Speech Therapy Department, Universidade Federal de Sao Paulo - Escola Paulista de Medicina.; 4 Master’s degree, physician of the Dermatology Department of the Universidade Federal de Sao Paulo/ Escola Paulista de Medicina.; 5 Specialist, master’s degree student, Universidade Federal de São Paulo - Escola Paulista de Medicina. Universidade Federal de Sao Paulo - Escola Paulista de Medicina.

**Keywords:** hearing, supression, vitiligo

## Abstract

Vitiligo is a skin disease characterized by absence of melanin due to melanocytes destruction. **Aim:** to study the incidence of hearing alterations in patients with vitiligo. **Method:** prospective audiological evaluation, transient-evoked otoacoustic emission recordings and study the effects of suppression in 24 patients with vitiligo. Their ages ranged from 15 to 45 years. **Results:** 21 patients (87.5%) had normal audiometry; 2 had unilateral hearing loss in the high frequencies and 1 had cochlear moderate hearing loss in the left ear. Of these 21 subjects, 66.7% had no otoacoustic emissions, suggesting cochlear dysfunction. Only 7 patients had otoacoustic emissions present in all frequencies (29.2%) and 17 (70.8%) did not have them, and the highest rate of no otoacoustic emissions happened in the right ear of males. Regarding the suppression study, 6 subjects failed, all of them were females, and their left ears were the most affected. **Conclusion:** the findings show that patients with vitiligo, particularly males, have a greater predisposition to cochlear dysfunction, especially in the right ear. As far as the suppression effect was concerned, there was a greater alteration in the female efferent system, particularly in the left ear. Hearing alterations did not vary as far as age is concerned, type of vitiligo and time of disease progression.

## INTRODUCTION

Vitiligo is an idiopathic acquired skin disease characterized by pearl-white patches of diverse shapes and sizes, caused by a loss of melanocytes (cells that synthesize melanin). It affects 1% of the general population, of which about 30% have a family history. It affects every race, and has no sex prevalence. The initial manifestations usually start at an age between 20 and 30 years, although the onset may occur in early infancy.^1^, ^2^

The etiology is unknown, but some theories have been used to explain the disease:

1) auto-immunity - due to frequent associations with other auto-immune disease such as thyroiditis and type I diabetes, the presence of anti-melanocyte antibodies, and a response to immunosuppressant therapy;
2) cytotoxicity - the possibility that metabolites formed during melanin synthesis may destroy melanocytes;
3) neural - chemical mediators released at nerve endings might destroy melanocytes or inhibit melanin production;
4) free radicals - excess free radicals might be toxic for melanocytes;
5) convergent - a combination of these theories.^2^ Vitiligo may be classified as localized, segmental, generalized and universal, according to the area of pigmentation loss.^2^

Alphonse Corti (1831) was the first researcher to mention the presence of pigment cells in the inner ear.^3^ There are many melanocytes in the human cochlea, particularly in the modiolus, in the osseous spiral lamina, in Reissner’s membrane and in the vascular stria; melanocytes are found especially in highly vascularized areas of apparently important secretory or metabolic function.^3^, ^4^, ^5^ Although its exact role - and that of melanin - remains unknown, it is probable that they have a vasomotor function in the inner ear.^3^, ^4^ According to Savin (1965)^3^ cells containing pigments are partially or fully adhered to blood vessel walls, which are sites of intense metabolite exchanges. For this author, melanin facilitates the passage of substances from one side to the other, thus maintaining cell membrane equilibrium. Furthermore, melanin may have an important role in the inner ear, since hearing is affected in systemic disorders that affect pigmented areas (eyes, skin and hair) such as the Vogt-Koyanagi and Waardenburg syndromes.^3^

The inner ear contains many melanocytes, particularly in the basal area of the cochlea, which is responsible for high sound frequencies, those that are affected earlier by ototoxic drugs and intense noise exposure.^4^, ^6^, ^7^ Conlee et al. (1995) studied the effect of gentamicin in animals and concluded that melanin could inhibit the toxicity of these drugs by binding to them; melanin would thus have a protective role for the cochlea.^8^

Carvalho (2004) found that otoacoustic emissions and the high frequency sensitivity auditory function was affected negatively in patients with pigment disorders, such as vitiligo.^9^

Recent studies have suggested a direct relation between cochlear dysfunction and decreased amounts of melanin. It is thus thought that melanin has a protective role against harmful agents in the inner ear.^4^, ^5^, ^7^ Loss of melanocytes, resulting in decreased melanin production - as occurs in Vitiligo - could decrease cochlear health.

The clinical uses of research on otoacoustic emissions and the suppression effect of contralateral noise have grown in importance. These are early detection methods of changes in cochlear function, even before these changes manifest themselves in hearing; they are also fast and non-invasive methods. Additionally, Guedes et al. (2002)^10^ found that in normal individuals the amplitude of transient evoked otoacoustic emissions (TOAE) vary little in intra-subject tests and retest, which strengthens its use in monitoring the hearing before symptoms manifest themselves.

The purpose of this study was to verify whether fewer melanocytes altered cochlear function, by measuring and analyzing TOAE, and to verify the function of the medial olivocochlear efferent system, by studying the effect of TOAE suppression in subjects with pigment disorders such as vitiligo.

## METHOD

The Research Ethics Committee approved this study (number 1065/06), which was undertaken at the outpatient unit of the Disciplina de Distúrbios de Audição (Hearing Disorders Discipline), Departamento de Fonoaudiologia (Speech Therapy Department), Universidade Federal de Sao Paulo (Sao Paulo Federal University).

All participants signed a free informed consent form. The sample in this study consisted of 24 subjects with vitiligo referred to the dermatology department of a university hospital. There were 15 female subjects (8 with generalized type vitiligo and 7 with localized type vitiligo) and 9 male subjects (5 with generalized type vitiligo and 4 with localized type vitiligo). The age ranged from 15 to 45 years (mean age - 31.29 years), and the mean duration of the disease was 9.23 years. The inclusion criteria were as follows: no history of otological diseases, no tinnitus, no having been subjected to intense noise, no past use of ototoxic drugs, and no family history of hearing loss. All patients were being treated dermatologically.

The clinical history was taken from each participant to establish the inclusion criteria; the external acoustic meatus was inspected to check for conditions that might interfere with the tests and the full audiological assessment. Testing comprised pure tone audiometry (MAICO 41 audiometer with TDH 39 earphones and an MX 41 pad - ANSI-69 standard) at 250 Hz, 500 Hz, 1 KHz, 2 KHz, 3 KHz, 4 KHz, 6 KHz, and 8 KHz in an acoustically treated test suite; normalcy was based on the criteria of the American Speech-language-hearing Association (ASHA, 1978)^11^ for thresholds below or equal to 20 dBHL, and conventional logoaudiometry (the speech recognition score - SRS, and the speech reception threshold - SRT). Normal result criteria adopted in this study were 92 to 100% of right answer in the SRS and intensities from 0 to 5dBHL over SRT thresholds.

Acoustic immittance tests consisted of tympanometry and investigation of the contralateral stapedian reflex (Interacoustics model AT - 235 middle ear analyzer); normal criteria were an A-type curve and contralateral stapedian muscle reflexes present and with intensities of 70 to 90 dBHL over the threshold.

Finally, patients underwent otoacoustic emissions testing (Otodynamics, London, ILO 96 Cochlear Emissions Analyzer) with a non-linear click at 80 microsecond regular pulses (duration of stimulus), a 50 cycle-per-second repetition frequency, and an 80 dBpeSPL (sound pressure equivalent peak) with 3 dBSPL, at a 6 000 Hz bandwidth and a 20 millisecond window. A series of 260 stimuli in blocks of four clicks in each test were presented. Contralateral acoustic stimulation was done with continuous white noise transmitted at an intensity of 50 dBSPL by a TDH 39 earphone to check for the absence or presence of otoacoustic emissions suppression.

The following order of tests was used in this study: TOAE in the right ear with no noise; TOAE in the right ear with contralateral noise; TOAE in the left ear with no noise; TOAE in the left ear with contralateral noise.

The normalcy criterion was the presence of TOAE 3 dB over noise in each frequency range (1 KHz, 1.5 KHz, 2 KHz, 3 KHz, and 4 KHz) with reproducibility equal to or over 70% and probe stability equal to or higher than 70%.

The suppression value of the olivocochlear system is given by the difference between values gathered with and with no contralateral stimulation for each ear; this value defines whether there is suppression of the amplitude of emissions or not.^12^, ^13^, ^14^ A suppression effect was considered as present when the TOAE amplitudes decreased by at least 0.5 dBSPL in the presence of contralateral noise. According to Collet et al. (1992), a 0.5 to 1.0 dB suppression effect reveals that the medial olivocochlear system is intact.^15^ It was considered as absent if the was no such decrease (zero or negative difference).^13^, ^14^

The significance value (p) was 5% (0.050) for the statistical tests and data analysis. The Fisher’s statistics adjusted chi-square test was applied to verify possible differences among categories in each case.

## RESULTS

Twenty-one (87.5%) of 24 patients with vitiligo (13 of the generalized type and 11 of the localized type) had normal pure tone audiometries, two had unilateral hearing loss (one in the right ear and one in the left ear) between 3 000 Hz and 8 000 Hz, and one had moderate cochlear loss in the left ear; thus, 12.5% of patients had hearing loss. Fourteen (66.7%) of 21 patients with normal audiometries presented partial (at 4 KHz) or absent TOAE, suggesting cochlear dysfunction. There were no sex-related statistically significant differences in conventional audiometry. Emissions were present at all frequencies in only seven patients (29.2%), and absent in 17 patients (70.8%), as shown in Table 1.

**Table 1:** Occurrence of hearing loss and transient otoacoustic emissions according to sex.

	Normal audiometry	Hearing loss	TOAE presente	TOAE absent
Male	8 (88,9%)	1 (11,1%)	1 (11,1%)	8 (88,8%)
Female	13 (86,7%)	2 (13,3%)	6 (40%)	9 (60%)
	21 (87,5%)	3 (12,5%)	7 (29,2%)	17 (70,8%)

Eleven (64.7%) of 17 patients in which TOAE were absent had bilateral failure, and six (35.2%) had unilateral failure. Four (36.3%) of 11 subjects with bilateral failure presented these failures at all frequency bands, and seven subjects (63.6%) had failure only at high frequencies (3 KHz and 4 KHz). Two female patients of six subjects with unilateral failure presented failure at all frequency bands in the left ear, and four patients had failure only at 4 000 Hz, of which three failed in the left ear.

There were no statistically significant differences in conventional audiometry as related to the type of vitiligo (p=0.566).

Altered emissions were more frequent in males compared to females (77.8% in which TOAE were absent), a difference that was statistically significant only in the right ear (p=0.035).

There were no statistically significant differences in the occurrence of TOAE in relation to the type of vitiligo (p=0.648), the duration of disease (p=0.406), or age (p=0.510), as shown in Tables 2 and 3.

**Table 2:** Occurrence of otoacoustic emissions in relation to the duration of disease.

	Present	Absent	
1 a 4 years	2 (18,2%)	9 (81,8%)	11
4 a 10 years	1 (16,7%)	5 (83,3%)	6
> 10 years	3 (42,9%)	4 (57,1%)	7
	6 (25%)	18 (75%)	24

**Table 3:** Occurrence of otoacoustic emissions in relation to the age of patients.

	Present	Absent	
15 a 30 years	2 (22,2%)	7 (77,7%)	9
30 a 43 years	5 (33,3%)	10 (66,7%)	15
	7 (29,2%)	17 (70,8%)	24

Suppression was investigated in 18 cases with bilateral emissions of the 24 sample subjects. There was bilateral suppression in 12 cases (66.6%), and absence of suppression in six cases (33.3%); all of these patients were female, with a statistically significant difference compared to males (p=0.034).

The two cases with unilateral TOAE (right ear only) were also taken into account in the assessment of the suppression effect per ear. The findings revealed that the left ear was more harmed; this difference in the occurrence of suppression relative to the side was statistically significant (p=0.024). Occurrence in the left ear (66.7%) was lower compared to the right ear (95%). The type of vitiligo did not affect the occurrence of suppression.

## DISCUSSION

We found in this study that there was a 12.5% hearing loss percentage in conventional pure tone audiometry. In studies on this theme, the evaluation of hearing revealed similar results (decreased hearing).

Tosti et al. (1987)^16^ found hearing loss in 16% of subjects with vitiligo; they raised the hypothesis that part of the melanocytes was injured by auto-immunity due to vitiligo. Auto-immunity is one of the accepted etiological theories of vitiligo.

Sharma et al. (2004)^17^ found that 18.9% of vitiligo patients had hearing loss at 2 000 Hz to 4 000 Hz, all of which were bilateral in a sample comprising 180 subjects.

Aydogan et al. (2005)^18^ assessed the audiological thresholds between 250 and 8 000 Hz and the electrophysiological potentials in 57 subjects with vitiligo and 50 healthy subjects. These authors found that 14% of vitiligo patients presented mild sensorineural dysacusis, of which six had bilateral hearing loss and two had unilateral hearing loss. They concluded that their findings, together with other published data, suggested that melanin might have an important role in establishing and/or maintaining the structure and function of the auditory system and in modulating the transduction of auditory stimuli in the inner ear.

Carvalho M. (2004)^9^ found seven patients (23.3%), in a sample of 30 vitiligo subjects, with thresholds above 25 dB in conventional audiometry. However, when compared with the control group, the authors found statistically significant differences only in high frequency audiometry.

Ardic et al. (1998)^7^ studied 29 subjects with vitiligo compared with a 41-subject control group, applying audiometry from 250 Hz to 16 KHz. This author found pure tone thresholds between 4 000 Hz and 10 000 Hz in the vitiligo group, which was significantly worse compared with the control group. Male patients presented hearing loss at higher frequency ranges than female patients, which was statistically significant. They concluded that vitiligo is a significant hearing loss factor, and that males are more vulnerable than females.

Our findings in the present study (hearing loss in 12.5% of subjects) are similar to other published results.^7^, ^9^, ^16^, ^17^, ^18^ Our study thus strengthens the hypothesis that vitiligo is a significant factor for altered cochlear function, and that melanin may in fact have an important role in cell metabolism, facilitating substance exchanges and maintaining endolymph, perilymph and ionic balance.^19^

Our findings also show that TOAE are a sensitive test for detecting cochlear dysfunction before symptoms become manifest, as TOAE were absent in 66.7% of subjects with normal audiometries. Carvalho M. (2004) concluded that emissions (distortion product, or DPOAE) and high frequency audiometry are sensitive tests for identifying cochlear dysfunction in vitiligo subjects.^9^ Our findings support this statement, adding that conventional audiometry with TOAE testing are reliable tests for the early detection of cochlear dysfunction.

Additionally in our study, we found increased auditory vulnerability in male subjects, as demonstrated by absent TOAE; this finding is similar to Ardic et al.’s results (1998).^7^ TOAE suppression studies have shown a more significant absence of suppression in females, showing altered auditory efferent pathways, specifically in the medial olivocochlear tract.

We found no published papers on the use of TOAE and the suppression effect in vitiligo.

There were no statistically significant differences in the present study related to the duration of vitiligo, or to age. Other published papers have also found no statistically significant differences related to the duration of the disease or age, compared to controls.^18^

Patients with suspected retrocochlear dysfunction due to lack of suppression were referred to an Otorhinolaryngologist and for brainstem audiometry.

We believe that all vitiligo patients required routine monitoring and audiological assessments by specialists for early identification and monitoring of changes as the disease progresses.

## CONCLUSION

Vitiligo patients appear to be more predisposed to cochlear dysfunction, as shown by the absence of otoacoustic emissions, although pure tone audiometry was within normal limits in most of the sample; this group was also more likely to develop changes in the medial olivocochlear efferent system.
